# The primary cilium at the helm: gatekeeper of TGF-β superfamily signaling in development, homeostasis, and disease

**DOI:** 10.1042/BST20253108

**Published:** 2026-06-03

**Authors:** Cristian Herrera-Cid, Oskar Kaaber Thomsen, Linéa Désarbre, Rylee Blair Marin, Canan Doganli, Lotte Bang Pedersen, Lars Allan Larsen, Søren Tvorup Christensen

**Affiliations:** 1 Department of Biology, Section for Cell Biology and Physiology, University of Copenhagen, Copenhagen, Denmark; 2 Department of Cellular and Molecular Medicine, University of Copenhagen, Copenhagen, Denmark; 3 College of Health Sciences, VinUniversity, Hanoi, Vietnam

**Keywords:** brain development, congenital heart disease, heart development, neurodevelopmental disorders, organ function, Primary cilia, TGF-β superfamily signaling, tissue homeostasis

## Abstract

The transforming growth factor-beta (TGF-β) superfamily is crucial for regulating cell proliferation, differentiation, migration, and tissue homeostasis, and plays a central role in embryonic development and function of tissues and organs. Dysregulation of these pathways contributes to a broad spectrum of diseases, including cancer, fibrosis, and developmental disorders. The present review explores how the primary cilium, a specialized signaling organelle, orchestrates TGF-β superfamily signaling, with a focus on emerging evidence of its role in heart and brain development, as well as in tissue homeostasis.

## Structural and signaling principles of the primary cilium

The primary cilium is a specialized microtubule-based organelle that functions as a cellular antenna for sensing and transducing extracellular cues [1,2]. Present on the surface of most vertebrate cell types, it has emerged as a central hub for coordinating developmental signaling and maintaining tissue homeostasis [1,2]. During embryogenesis, primary cilia are found in diverse cell populations, from epiblast cells at early developmental stages [3] to later derivatives such as neural [4,5] and cardiac stem cells [6,7]. However, despite advances in imaging and molecular markers, systematically identifying all ciliated cell types during development remains challenging.

**Figure 1:**
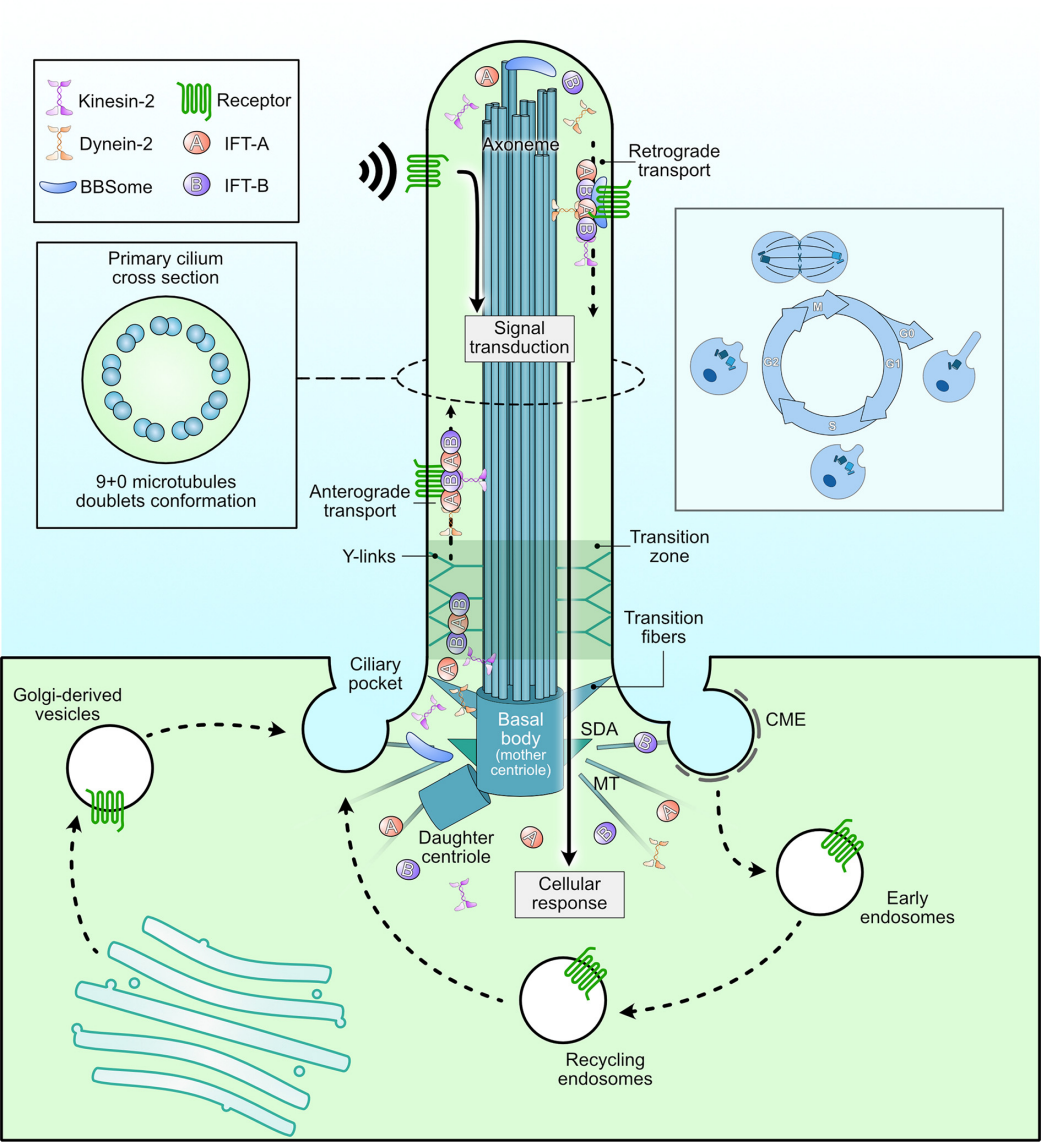
Overview of the primary cilium The primary cilium is a non-motile organelle that protrudes as a single projection from the centrosomal mother centriole (basal body) during growth arrest. The main structure consists of a microtubule (MT)-based axoneme arranged as ‘9+0’ peripheral doublets. The transition zone between the basal body and transition fibers from the basal body gates the import and export of ciliary proteins, ensuring organelle compartmentalization. Intraflagellar transport (IFT) shuttle proteins, including membrane receptors, into the cilium for signaling or out for recycling or degradation. The presence of primary cilia is tightly coordinated with the cell cycle. Ciliogenesis occurs during G0/G1, whereas the cilium is disassembled as cells re-enter the cell cycle. Abbreviations: CME: clathrin-mediated endocytosis; SDA: subdistal appendage.

Structurally, the primary cilium is a solitary, non-motile projection that extends from the cell surface and originates from the basal body, which is derived from the mother centriole of the centrosome (Figure 1). Its ability to integrate spatial and temporal signaling cues relies on a highly organized architecture and tightly regulated assembly-disassembly cycles that are coordinated with the cell cycle (Figure 1). In addition, selective trafficking of signaling components into and out of the ciliary compartment enables the primary cilium to function as a specialized platform for multiple signaling pathways [1,2,8].

The importance of this organelle is highlighted by genetic studies demonstrating that complete loss of cilia results in embryonic lethality in vertebrate models. In contrast, partial defects in ciliary structure or function cause a diverse group of disorders collectively termed ciliopathies [1,9–11]. These conditions affect multiple organs and commonly include obesity, retinal degeneration and blindness, renal cyst formation and fibrosis, polydactyly, craniofacial and skeletal abnormalities, as well as neurological and congenital heart defects [1,9–11]. Because ciliopathies lack effective therapies [1,12], there is a critical need to advance our understanding of the spatiotemporal regulation of primary cilia within defined cell types and tissue contexts and how these dynamics control signaling pathways during organogenesis and the maintenance of tissue homeostasis.

## Assembly and compartmentalization of the primary cilium for signaling

The primary cilium contains a microtubule-based axoneme surrounded by a specialized membrane enriched in signaling receptors, ion channels, and distinct lipids that support its sensory functions [2,13]. The axoneme displays a characteristic 9+0 microtubule arrangement, consisting of nine outer microtubule doublets without a central pair, which distinguishes it from the 9+2 architecture of motile cilia [1]. At the base of the cilium lies the transition zone, a structural and functional gate that regulates the selective entry and exit of proteins, thereby maintaining the distinct molecular composition of the ciliary compartment [1] (Figure 1). Adjacent to this region is a specialized periciliary membrane domain that separates the ciliary and plasma membranes and functions as an active site for endocytic and exocytic trafficking associated with ciliary signaling. This region is often organized into a plasma membrane invagination known as the ciliary pocket, whose size can vary depending on cell type [14]. For example, the pocket is mostly absent in kidney tubular epithelial cell types while very prominent in most mesenchymal cell types [14,15].

Ciliogenesis typically occurs during the G0/G1 phase, i.e., in growth-arrested cells. The process begins with docking of vesicles at the distal appendages of the mother centriole [1], followed by elongation of the axoneme through IFT, a bidirectional trafficking system essential for ciliary assembly and maintenance [16–18]. The IFT machinery consists of IFT-A and IFT-B complexes, which bind ciliary cargo directly or via adaptor proteins and move along axonemal microtubules through the action of kinesin-2 motors (anterograde transport) and cytoplasmic dynein-2 (retrograde transport) (Figure 1) [17,18]. This transport system mediates the dynamic movement of signaling proteins, including transmembrane receptors such as specific G protein-coupled receptors (GPCRs). For example, the adaptor protein Tubby-like protein 3 (TULP3) facilitates ciliary import of GPCRs through interactions with the IFT-A complex [19,20]. Conversely, the BBSome, an octameric cargo adaptor associated with IFT-B, facilitates the export of membrane proteins from the cilium; mutations in its components lead to Bardet–Biedl syndrome (BBS), a ciliopathy characterized by features such as obesity, retinal degeneration, polydactyly, neurodevelopmental impairment, and renal abnormalities [21–25]. Although loss of individual BBS proteins does not necessarily prevent ciliogenesis [26], it disrupts the composition of the ciliary membrane and the localization of signaling receptors, thereby altering cellular responsiveness to extracellular cues [1,11,26–28].

Disassembly of the primary cilium is typically initiated during cell cycle entry and progresses toward S phase, allowing duplicated centrosomes to participate in mitosis [29]. Prominent examples of ciliary disassembly regulators include the scaffolding protein HEF1, also known as NEDD9, and Aurora kinase A (AURKA), which phosphorylates HDAC6 to deacetylate axonemal tubulin, destabilize microtubules, and drive ciliary resorption [30]. Alternatively, cilia can be disassembled through ciliary decapitation or shedding. In ciliary decapitation, AURKA-mediated phosphorylation of inositol polyphosphate 5-phosphatase (INPP5E), a phosphatidylinositol 3,4,5-triphosphate and phosphatidylinositol 4,5-biphosphate 5-phosphatase crucial for regulating the primary ciliary membrane composition [31], promotes actin-dependent excision of the distal tip [32,33]. In contrast, ciliary shedding entails the rapid release of most of the ciliary structure [34]. Defects in the regulation of ciliary disassembly have important functional consequences, including disrupted cellular signaling and impaired cell cycle control, and have been linked to diseases such as neurodevelopmental disorders and cancer [8,35,36].

Primary cilia act as signaling hubs for multiple conserved pathways, including Hedgehog (Hh), WNT, Notch, GPCR, and receptor tyrosine kinase signaling such as platelet-derived growth factor receptor alpha (PDGFRα) and fibroblast growth factor receptor (FGFR), as well as members of the TGF-β superfamily [2]. A defining feature of this organization is the biochemical specialization of the ciliary compartment, which concentrates receptors and signaling intermediates to achieve higher local effective concentrations than in the surrounding cytoplasm [1,8,37,38] and enhances both the efficiency and specificity of signal transduction compared with signaling at the bulk plasma membrane [29,36,39].

Among these pathways, Hh signaling is the best-characterized example of obligate cilium-dependent signaling in vertebrates, where signaling is strictly controlled by the regulated, temporal trafficking of the core components patched1 and smoothened into and out of the ciliary membrane upon ligand stimulation. This dynamic relocalization governs pathway activation and ultimately controls GLI transcription factor activity and downstream gene expression [2,40,41].

Other pathways, including PDGFRα [42,43], FGFR [44], and TGF-β superfamily [45–49] signaling, also exhibit ciliary enrichment of specific pathway components and may be similarly regulated by trafficking-dependent mechanisms within the primary cilium. However, for these pathways, the molecular mechanisms governing ciliary targeting and retention are less well defined than for Hh signaling, and the degree to which signaling output depends on ciliary trafficking and localization can vary between pathways and cell types, reflecting differences in their requirement for the primary cilium in physiological and developmental contexts [1].

## Key mechanisms of TGF-β superfamily signaling

Accumulating evidence underscores the pivotal role of TGF-β signaling in organogenesis and tissue homeostasis. These processes are orchestrated through tightly coordinated regulation of cell proliferation, differentiation, and migration, highlighting the necessity of precise temporal and spatial control during organ development [50].

The human TGF-β superfamily comprises over 33 ligands that govern the development and function of diverse tissues and organs [50,51]. These ligands are grouped into several subfamilies, including TGF-βs, bone morphogenetic proteins (BMPs), nodal, activins and inhibins, anti-Müllerian hormone, and growth and differentiation factors (GDFs) [51]. Signal transduction is mediated through heterotetrameric receptor complexes, composed of two type I (RI) and two type II (RII) serine/threonine kinase receptors, which initiate distinct downstream cascades classified as canonical (SMAD-dependent) or non-canonical (SMAD-independent) pathways [50]. Additionally, type III receptors, also known as betaglycan, can function as co-receptors for TGF-β superfamily ligands, modulating ligand availability and facilitating access to RI and RII [52].

**Figure 2:**
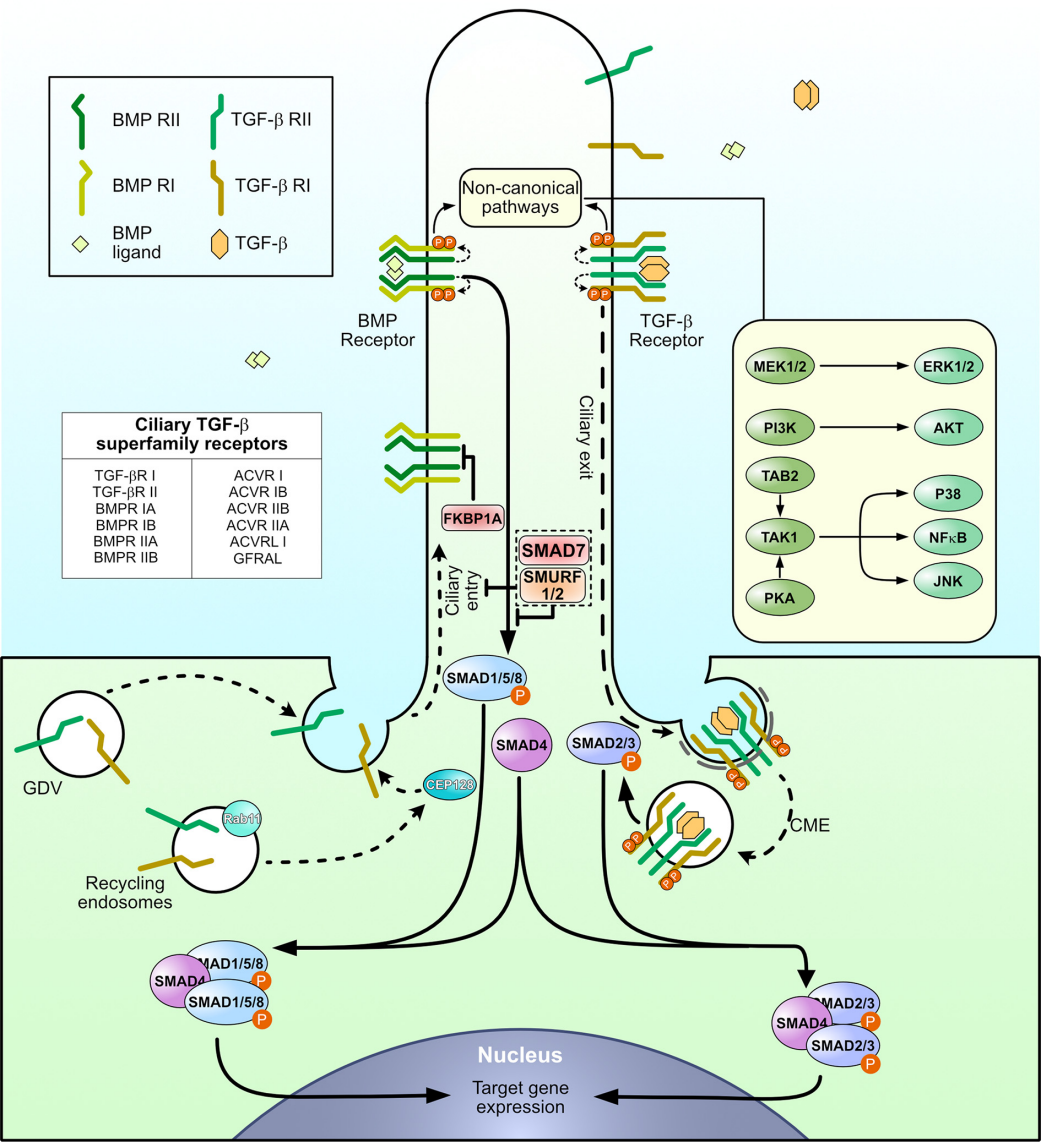
TGF-β superfamily signaling is coordinated by the primary cilium Receptors for TGF-β, BMP, and activin are enriched at the primary cilium. Ligand binding induces assembly of a heterotetrameric complex in which two type II (RII) receptors form complex with and subsequently phosphorylate two type I (RI) receptors. Activated receptors exit the cilium via CME and enter early endosomes, leading to R-SMAD phosphorylation and formation of R-SMAD/SMAD4 complexes that translocate to the nucleus. Inhibitory SMADs, such as SMAD7, in addition to other regulators, SMURF1/2 and FKBP1A, suppress canonical signaling. TGF-β and BMP receptors also activate non-canonical pathways within the cilium. Receptor delivery to the cilium relies on Golgi-derived vesicles (GDVs) and CEP128-dependent Rab11-positive recycling endosomes.

In the canonical pathway, ligand-bound receptor complexes may undergo clathrin-mediated endocytosis (CME) [53–55], facilitating recruitment of receptor-regulated SMADs (R-SMADs) to the receptor complex and their subsequent phosphorylation in early endosomes [56]. In contrast, caveolin-mediated endocytosis (CvME) [53–55] may attenuate signaling by reducing R-SMAD phosphorylation [57] and promoting receptor degradation via the inhibitor SMAD (I-SMAD), SMAD7, in conjunction with the HECT-family E3 ubiquitin ligase, SMURF2 [58]. Reciprocal neddylation and ubiquitination of receptors direct them preferentially toward CME and CvME, respectively [59], highlighting a critical regulatory checkpoint in TGF-β signaling (Figure 2).

Ligand specificity dictates R-SMAD activation: TGF-βs and Activins predominantly engage SMAD2/3, whereas BMPs signal via SMAD1/5/8 [51]. Certain GDFs can activate both branches [50,51]. I-SMADs, including SMAD6 and SMAD7, dampen signaling by either directly antagonizing R-SMAD activity or recruiting E3 ubiquitin ligases, such as SMURF1/2 and NEDD4L, to target receptors for degradation [60,61]. Additional modulators, such as FKBP1A (FKBP12), bind constitutively to type I receptors to suppress downstream signaling until TGF-βRI is phosphorylated by TGF-βRII, releasing this inhibition [62] (Figure 2).

Beyond SMADs, TGF-β superfamily signaling engages non-canonical pathways, including PI3K/AKT, Rho-family GTPases, ERK1/2, and TGF-β-activated kinase 1 (TAK1; also known as MAP3K7) [50]. TAK1 subsequently activates downstream MAPKs, such as p38 and JNK1/2, as well as the NF-κB pathway [50]. Activation of these pathways can in turn modulate R-SMAD activity through direct cross-talk [63,64]. These non-canonical pathways thus provide additional layers of regulation and context-dependent modulation of cellular responses (Figure 2).

Increasing evidence indicates that multiple TGF-β superfamily receptors localize to primary cilia, including ACVRI/ALK2, ACVRIIA/B, BMPRI/ALK3, BMPRII, TGF-βRI/ALK5, and TGF-βRII [45,47–49,65]. Regulatory components such as FKBP1A, SMURF1, and SMAD1–7 have also been shown to localize to primary cilia in diverse cell types, enabling fine-tuning of signaling outputs as will be discussed below [45,65–67] (Figure 2). The primary cilium may thus serve as a critical nexus for TGF-β superfamily signaling, with emerging roles in coordinating heart and brain development and maintaining tissue homeostasis.

## Primary cilia and TGF-β superfamily signaling in heart development

During embryogenesis, the heart is the first organ to form [68], and its development relies on precise coordination of morphogenetic processes and signaling pathways [68,69]. During gastrulation, around week 3 in humans, when the primary germ layers are established and the body’s main axes are defined, epithelial-mesenchymal transition (EMT) drives germ layer formation. This process generates cardiac precursors that assemble the cardiac crescent, a layer positioned between the ventral and dorsal endoderm, comprising the first heart field (FHF) and second heart field (SHF) [68]. Expansion and midline fusion of the cardiac crescent generate the linear heart tube, which subsequently undergoes looping and chamber ballooning to establish the early heart architecture.

The differentiation and proliferation of cardiac progenitors within the FHF and SHF require tightly coordinated cell cycle regulation, a process in which the primary cilium emerges as a critical modulator [69]. Several ciliary signaling pathways have been shown to influence cell cycle progression, such as lysophosphatidic acid signaling, which promotes ciliary disassembly, thereby facilitating cell cycle re-entry and proliferation [70]. TGF-β superfamily signaling is likewise implicated in regulating cell cycle dynamics and cardiogenesis [71], although the mechanism linking TGF-β signaling to ciliary control of cardiac progenitor proliferation remains unclear.

Following cardiac tube formation during early gastrulation, motile cilia in the embryonic node of mammals, also known as Kupffer’s vesicle in zebrafish, generate a leftward fluid flow detected by surrounding primary crown cilia. This mechanosensory input induces asymmetric ciliary Ca^2^⁺ influx and activates Nodal signaling on the left side, initiating left–right (LR) symmetry breaking. Impaired nodal cilia motility can therefore disrupt this process, leading to heterotaxy, which is frequently associated with abnormal cardiac looping, a critical step in four-chambered heart formation [72–75]. Valve morphogenesis begins with the development of cardiac cushions—localized extracellular matrix swellings. Atrioventricular cushions arise from endothelial cells undergoing endothelial-to-mesenchymal transition [76], while muscular septation between atria and ventricles is supported by the dorsal mesenchymal protrusion derived from the SHF [69]. Concurrently, the outflow tract remodels, and the epicardium forms as the proepicardium envelops the heart [77].

Many of these processes, including EMT and progenitor differentiation, are modulated by TGF-β superfamily signaling [78,79], and disruption of this pathway may lead to congenital heart defects (CHD) [80,81]. Emerging evidence positions the primary cilium as a coordinator of both canonical and non-canonical TGF-β pathways, with ciliary defects and altered TGF-β signaling dynamics contributing to CHD, a frequent manifestation of several ciliopathies [46,65,72,82].

**Figure 3:**
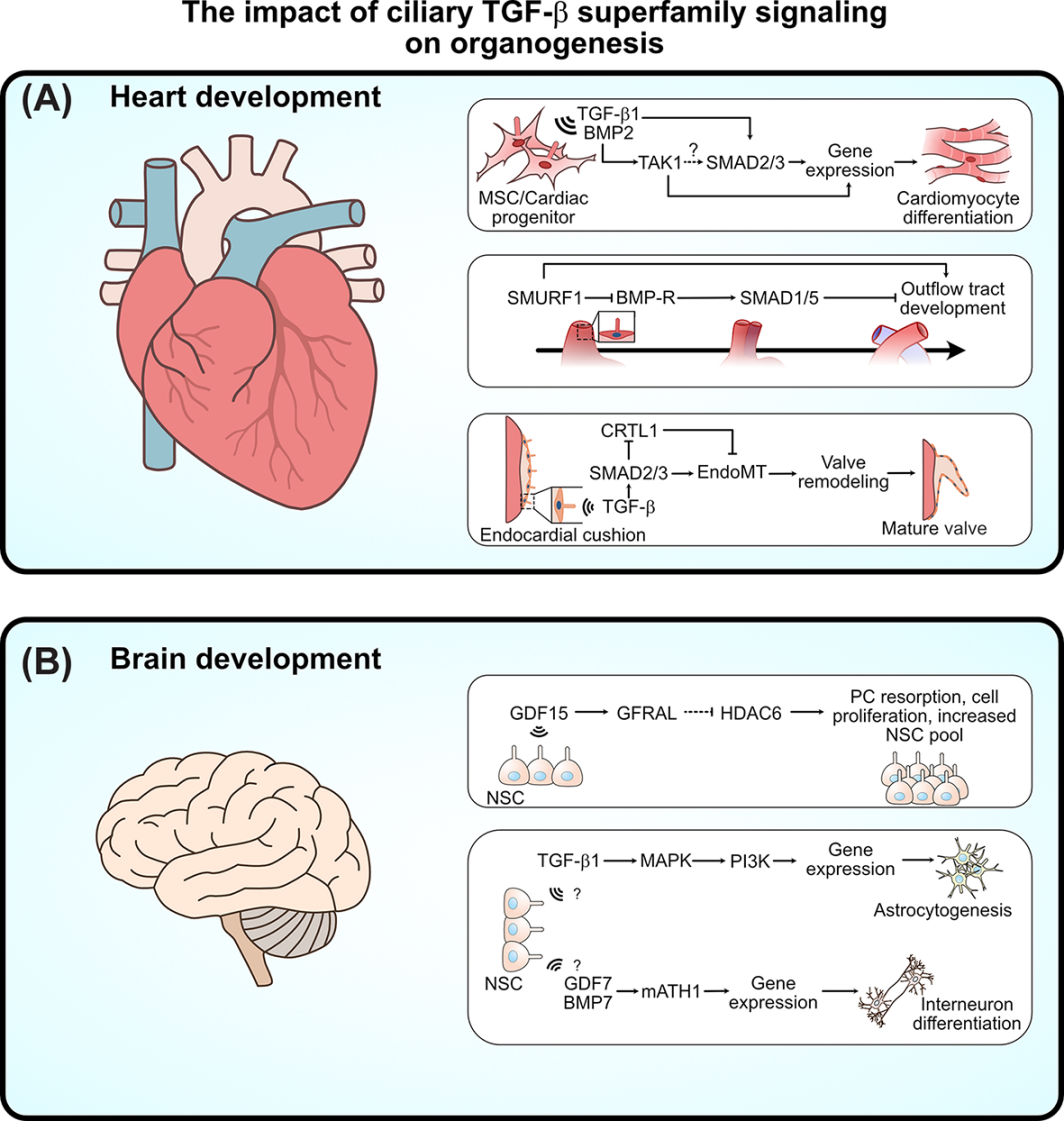
Ciliary TGF-β superfamily signaling in heart and brain development (**A**) In *in vitro* studies, ciliary TGF-β superfamily signaling regulates mesenchymal stem cell (MSC) differentiation into cardiomyocytes via TAK1 and SMAD2/3 signaling. TAK1 may further promote SMAD2/3 activity via phosphorylations in the SMAD linker region [87]. Inhibition of BMPR-mediated SMAD1/5 signaling by SMURF1 is necessary to develop the outflow tract. While TGF-β-dependent SMAD2/3 signaling is required for proper heart valve formation through promotion of endothelial-mesenchymal transition (EndoMT) in the endocardial cushions. (**B**) During brain development, ciliary TGF-β superfamily regulates the proliferation of neural stem cells (NSCs) through ciliary GDF15-GFRAL signaling and downstream HDAC6 inhibition, which limits ciliary resorption and cell cycle progression. TGF-β superfamily signaling also regulates the differentiation of astrocytes and interneurons through MAPK-PI3K and mATH1 activation, respectively, though here potential ciliary involvement remains unresolved.

A CHD-cilium screen identified the gene encoding TAB1 (TAK1 Binding Protein 1) [50], a component of the TAB1-TAB2-TAK1 signalosome in the non-canonical branch of TGF-β signaling. Genetic studies further implicated TAB2 as a CHD gene, with copy number variants identified in patients and heart defects observed in *tab2* knockout zebrafish [83,84]. TAK1 is essential for cardiac differentiation in P19.CL6 stem cells [84,85], and both TAK1 and TAB2 localize to primary cilia, where TAB2 accumulates and TAK1 becomes phosphorylated at two activation sites at the ciliary base in a differentiation-dependent manner, modulating downstream JNK signaling [84]. Canonical TGF-β signaling is similarly coordinated at the cilium via CME of activated receptors at the ciliary pocket, regulating cardiomyogenesis in P19.CL6 and human embryonic stem cells (hESCs) [49]. Supporting the centrality of ciliary TGF-β signaling in heart development, whole-exome sequencing of 218 C57/BL6 mouse embryos with CHD identified 34 cilia-related genes associated with cardiac malformations, six of which, *Cfc1*, *Ltbp1*, *Megf8*, *Pcsk5*, *Smad6*, and *Tab1*, are directly involved in TGF-β superfamily signaling [86]. Together, these findings establish both canonical and non-canonical ciliary TGF-β superfamily signaling as essential regulators of cardiac differentiation and highlight the primary cilium as a hub linking TGF-β superfamily signaling to CHD (Figure 3A).

TGF-β signaling is a central orchestrator of EMT during valve formation. TGF-β2 is required for cushion mesenchyme differentiation and subsequent valve remodeling [79], a process that has recently been shown to depend on intact primary cilia [88]. Ciliary dynamics are further regulated by CYLD, a tumor-suppressor deubiquitinase, which promotes ciliary resorption by antagonizing HDAC6, thereby linking ciliary remodeling to TGF-β-mediated EMT during pulmonary fibrosis [89]. Disruption of ciliary function impairs valve morphogenesis, as evidenced by defective bicuspid valve formation [90], and SMURF knockout mice exhibit delayed outflow tract septation [65]. Loss of SMURF1 similarly accelerates cardiomyogenic differentiation in P19.CL6 stem cells, influencing SHF progenitors during hESC differentiation, likely through perturbed SMAD1/5/8 regulation of BMP signaling at the cilium [65] (Figure 3A).

Despite these advances, several processes remain to be linked to ciliary signaling. LR asymmetry at the node depends on a BMP4 gradient, but the role of ciliary signaling in this context is unclear [91]. Endothelial shear stress mechanotransduction is mediated via a ciliary β-arrestin-BMPRII module, with loss of β-arrestins impairing retinal vascularization, though its impact on cardiac vasculature remains unknown [47]. These findings underscore a central role of ciliary TGF-β signaling in heart development and highlight the need for further investigation of ciliary contributions during cardiogenesis.

## Primary cilia and TGF-β superfamily signaling in brain development

Neurodevelopment is a tightly coordinated process that begins with the formation of the neural plate, which folds into the neural tube through cellular movements such as convergence, extension, and apical constriction [92,93]. Following neural tube closure, the tube regionalizes into forebrain, midbrain, and hindbrain domains, driven by differential proliferation and spatial organization of neural progenitors. These domains give rise to structures including the telencephalon (cerebral cortex, basal ganglia), diencephalon, mesencephalon, metencephalon (pons, cerebellum), and myelencephalon [94]. Neural stem cells then divide and migrate from the ventricular zone to superficial layers along radial glia and extracellular matrix cues, establishing the brain’s layered architecture, especially in the cerebral cortex [36]. Upon reaching their destinations, neurons differentiate, extending axons and dendrites [95]. Synaptogenesis follows, forming synaptic contacts, which are later refined through activity-dependent stabilization or elimination, shaping functional neural circuits [95,96]. The primary cilium is central to these processes, as evidenced by the wide spectrum of neurological defects in ciliopathy patients, including epilepsy, cognitive impairments, and diverse central nervous system malformations [97,98], as well as emerging links to neurodegenerative diseases such as Alzheimer’s, Parkinson’s, and Huntington’s disease [98].

Despite growing recognition of the primary cilium as a signaling hub, our understanding of ciliary TGF-β superfamily signaling in neurodevelopment remains limited. Emerging evidence indicates that disruption of ciliary structure or function can cause premature differentiation or failure to properly exit the cell cycle, depleting neural progenitor pools and impairing brain development [99,100]. For instance, GDF15 regulates proliferation of neural stem cells through the primary cilium [101], restricting cell division in the ventricular-subventricular zone to control early corticogenesis. Mechanistically, activation of its ciliary receptor inhibits HDAC6, stabilizing and elongating the cilium, which slows the cell cycle and reduces neural stem cell proliferation [102] (Figure 3B).

However, how ciliary defects influence TGF-β signaling across the diverse developmental stages and cellular processes of the brain remains poorly understood, representing a major challenge in the field. Key open questions include the role of cilia in early neurulation, where precise temporal inhibition of TGF-β signals is required to initiate neural tube formation [103,104], followed by reactivation to drive neural tube closure, neural crest formation, and dorsoventral patterning, including roof and floor plate specification [105–109]. Disruption of these signaling events, as seen in Nodal pathway zebrafish mutants, results in ventral brain loss and incomplete neural tube closure [108]. Later in development, TGF-β signaling governs the maintenance and differentiation of radial glia, a critical neural stem cell population. For example, TGF-β1 treatment in mouse embryos increases astrocyte numbers in the cerebral cortex through non-canonical MAPK/PI3K pathways (Figure 3B) [110,111], while BMP family members, including BMP4, BMP7, and GDF7 (also known as BMP12), regulate roof plate development and dorsal interneuron differentiation by activating *Lim* and *Msx1* transcription factors [104,112–115]. Loss of the GDF7 ligand or BMP receptors disrupts *Wnt* and *Math1* expression, impairs dorsoventral patterning, and leads to selective interneuron loss (Figure 3B) [116,117].

Ciliary defects mirror many of these developmental abnormalities. Mice carrying loss-of-function alleles of IFT172 (BBS20), either through exon deletion [118,119] or a L1564P mutation [119], exhibit disrupted anterior mesendoderm formation, craniofacial malformations, failed cranial neural tube closure, and forebrain defects, including truncation of the telencephalon and diencephalon or holoprosencephaly. Mechanistically, truncation of the IFT172 U-Box domain, which may bind poly-ubiquitinated proteins, notably results in disproportional canonical and non-canonical TGF-β signal transduction output [120], suggesting a role for ciliary TGF-β signaling in the IFT172 mutation phenotype [120]. Similarly, conditional deletion of ARL13B, a GTPase critical for ciliary formation and maintenance [121–123], alters interneuronal cilia dynamics and morphology, leading to mislocalized neurons with reduced dendritic branching [124]. Together, these overlapping phenotypes suggest an intersection between ciliary function and TGF-β signaling in neurodevelopment; however, they may also reflect broader defects in additional signaling pathways. Notably, Hh signaling is significantly hampered in ARL13B-deficient cells and mice [123,125]. Further investigations are required to delineate the precise contributions of the ciliary TGF-β superfamily signaling landscape during neurodevelopment.

In conclusion, while parallels between TGF-β superfamily signaling and ciliary defects in neurodevelopment are apparent, understanding how pathway components integrate into the ciliary signaling network represents a major frontier. Addressing these gaps will not only elucidate fundamental mechanisms controlling neural progenitor proliferation, differentiation, and patterning but also provide unprecedented insight into the etiology of neurodevelopmental disorders. A systematic dissection of ciliary TGF-β superfamily signaling has the potential to reveal novel regulatory nodes, identify therapeutic targets for ciliopathies and related brain disorders, and ultimately transform our understanding of how complex neural circuits are established.

## Primary cilia and TGF-β signaling in bone development and repair

**Figure 4:**
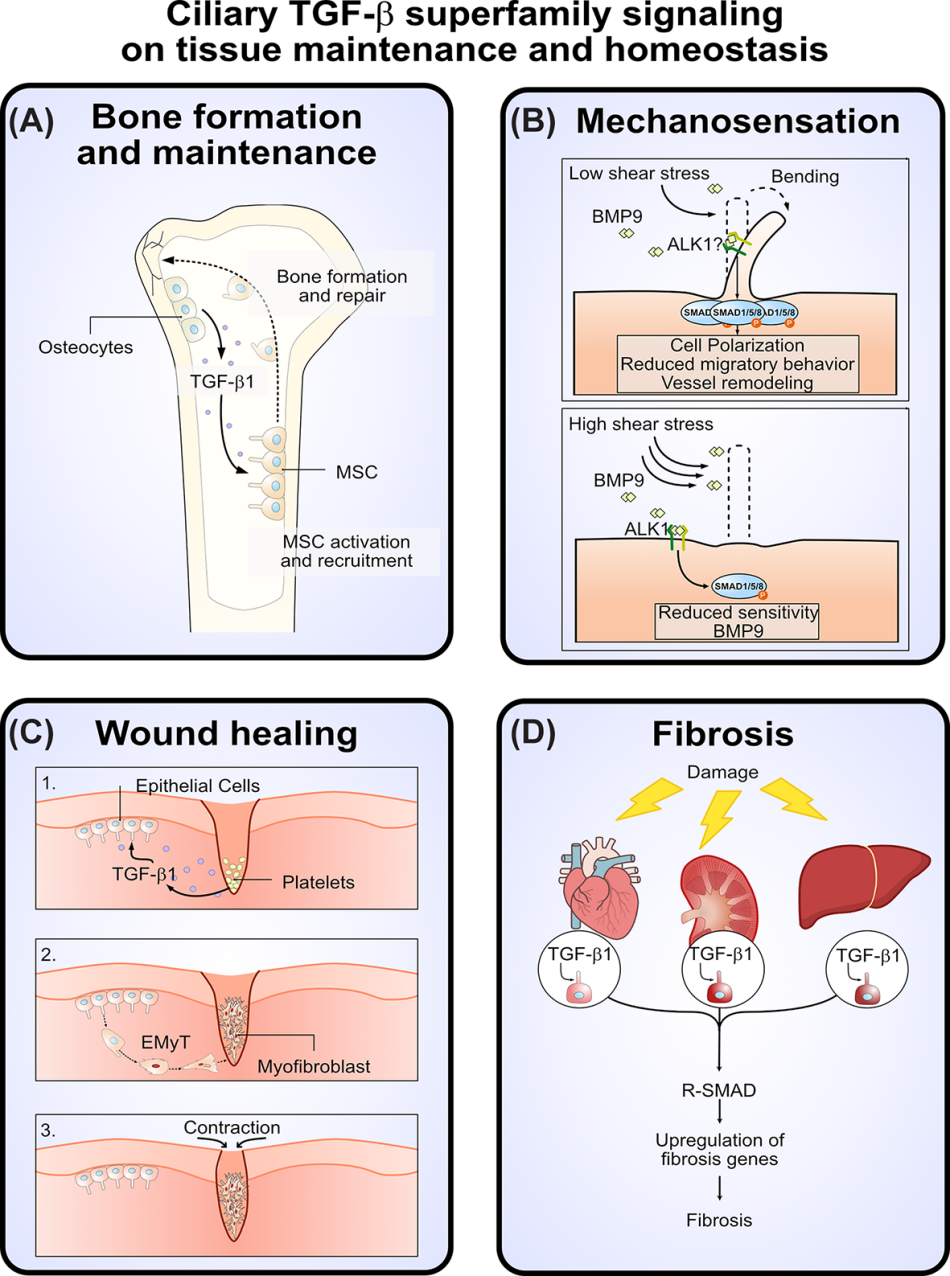
Ciliary TGF-β superfamily signaling in tissue maintenance and homeostasis (**A**) Ciliary TGF-β signaling is critical in bone development and maintenance. During bone formation and repair, TGF-β superfamily signaling promotes MSC recruitment, osteogenic differentiation, osteoblast activity, and ossification. (**B**) Ciliary BMP9 signaling is heightened in endothelial cells during low shear stress conditions, modifying the cellular response and gene expression in these cells. During high shear stress, the primary cilium is lost and BMP9 sensitivity is decreased [131]. (**C**) During tissue repair, ciliary TGF-β signaling regulates fibrosis and wound healing by promoting epithelial-to-myofibroblast transition (EMyT) of the surrounding epithelium. (**D**) After injury in the heart, kidney, and liver, ciliary TGF-β1-SMAD3/4 signaling controls fibrosis through the promotion of fibrotic gene expression, including collagen and fibronectin.

Ciliary TGF-β superfamily signaling also plays a central role in bone formation, homeostasis, and tissue repair. Human bone MSCs, multipotent progenitors that differentiate into osteoblasts and chondrocytes, rely on TGF-β1 signaling at the primary cilium for recruitment and osteogenic differentiation via SMAD3 activation, thereby supporting bone formation and repair [126]. Similarly, stem cells from exfoliated deciduous teeth are activated via ciliary activin and BMP signaling through ACVRI/ACVRIIA-mediated R-SMAD phosphorylation [45] (Figure 4A). Knockdown of IFT88, required for ciliogenesis [2], or ADP-ribosylation factor-like protein 3 (ARL3), essential for ciliary cargo transport and homeostasis [127–129], completely ablates this pathway [130].

Pathological hyperactivation of ciliary BMP signaling further illustrates its functional importance. The gain-of-function mutation in ACVRI (Q207D), associated with fibrodysplasia ossificans progressiva, localizes to the primary cilium and drives heterotopic ossification in mice by amplifying BMP signaling [45]. This aberrant ossification is rescued either by Cre-Lox-mediated deletion of IFT88 or ARL3 or by targeted ARL3 siRNA treatment [45]. Additionally, BMP receptor signaling at the cilium is essential for osteogenic differentiation and osteoblast maturation, as demonstrated under pulsed electromagnetic field stimulation [48]. Loss-of-function studies reinforce this concept: deletion of IFT80 in chondrocytes within bone fracture calluses impairs healing, eliminates primary cilia, reduces TGF-β1 and TGF-βRI expression, and decreases R-SMAD phosphorylation, indicating that cilia are crucial mediators of chondrocyte-driven TGF-β signaling during bone repair [132].

Primary cilia also function as mechanosensory hubs in bone and vascular tissues, linking TGF-β superfamily signaling to mechanical cues and injury repair. In the developing mouse retina, endothelial cilia enhance BMP9 sensitivity under low shear stress, stabilizing vessel connections and maintaining vascular patency [131]. Similarly, tensile strain in periodontal ligament cells up-regulates TGF-β ligands, receptors, and ciliary markers, accompanied by SMAD1/5/8 phosphorylation at the ciliary base, suggesting a mechanosensory role for ciliary TGF-β signaling [133] (Figure 4B). In musculoskeletal repair, loss of primary cilia impairs recruitment of Prrx1+ mesenchymal progenitors, disrupting tissue healing [124]. Flow-induced stimulation with TGF-β1 enhances progenitor migration *in vitro*, an effect abolished by knockdown of IFT88 or Pallidin, which are required for ciliary formation and trafficking [134,135].

## Ciliary TGF-β signaling in fibrosis

Wound healing frequently involves fibrotic remodeling, a process largely driven by increased TGF-β signaling [136]. Fibrosis typically arises from myofibroblasts generated through fibroblast-myofibroblast or epithelial-myofibroblast transition. During this process, epithelial cells lose E-cadherin-mediated cell-cell junctions and undergo morphological remodeling, which is accompanied by dynamic changes in primary cilia, including transient elongation followed by complete ciliary resorption (Figure 4C) [137]. These changes in ciliary structure appear to be partially regulated by TGF-β-dependent accumulation of OFD1, a protein localized at the distal end of the basal body that regulates processes such as ciliogenesis, EMT, and autophagy [138].

Recent evidence suggests that TGF-β signaling can influence ciliary dynamics through modulation of autophagy pathways. In serum-starved HK-2 cells, a human kidney proximal tubular epithelial cell line, TGF-β stimulation induces accumulation of OFD1 at the ciliary base, accompanied by increased LC3-II and p62 levels, indicating impaired autophagic flux [139]. These changes correlate with ciliary shortening and increased expression of fibrotic markers [139]. Inhibition of SIRT2, an NAD⁺-dependent deacetylase involved in stress responses and autophagy regulation, partially rescues these defects, suggesting that SIRT2 acts downstream of TGF-β signaling to regulate ciliary homeostasis and fibrosis-associated cellular remodeling [139]. However, whether these effects are mediated directly through ciliary TGF-β signaling remains to be clarified.

Evidence from cardiac fibroblasts further supports a functional relationship between primary cilia and TGF-β-driven fibrosis. TGF-β stimulates collagen biosynthesis, a hallmark of fibrotic remodeling, and this process depends on intact primary cilia, as knockdown of structural ciliary proteins abolishes TGF-β-induced collagen production [140]. *In vivo*, ciliated fibroblasts accumulate at sites of myocardial injury, whereas experimental promotion of ciliary disassembly through Ift88 knockdown enhances cardiac regeneration in mice, highlighting a complex interplay between ciliary signaling, fibrosis, and regenerative responses [72,140]. TGF-β signaling also contributes to fibrosis in other organs, including the kidney and liver, where elevated TGF-β expression and downstream SMAD3 and SMAD4 activity promote fibrotic progression (Figure 4D) [141,142].

Clinical observations provide further evidence linking ciliary dysfunction and TGF-β signaling to fibrosis. Several ciliopathies are characterized by progressive organ fibrosis, including nephronophthisis (NPHP), which primarily affects the kidneys and occasionally the liver [143]. In NPHP1 knockdown and knockout models, increased TGF-β signaling is observed, and overexpression of the inhibitory SMAD7 restores signaling balance and reduces fibrotic responses [144], suggesting that dysregulated TGF-β signaling contributes to fibrosis associated with ciliary dysfunction.

Collectively, these findings highlight an emerging but still incompletely understood role for primary cilia in regulating TGF-β-driven fibrotic responses. By modulating signaling dynamics, autophagy pathways, and cellular differentiation programs, the primary cilium may act as a critical integrator of TGF-β signaling during wound healing and tissue remodeling.

## Outlook

The studies discussed in the present review highlight the role of the TGF-β superfamily in coordinating key processes during organogenesis, including cell fate specification, proliferation, migration, and tissue patterning. Increasing evidence further positions the primary cilium as a critical regulatory platform that spatially and temporally organizes TGF-β signaling, enabling cells to integrate morphogenetic cues during development. Through the cilium, TGF-β pathways may contribute to the precise control of organ development, including cardiac, neural, and skeletal development, while also influencing tissue maintenance and repair later in life.

TGF-β superfamily signaling has emerged as an important therapeutic target in multiple diseases, particularly within the cardiovascular system [145]. In the context of acute myocardial infarction, TGF-β1 exhibits a biphasic role: early signaling dampens inflammatory responses and limits initial tissue damage, whereas prolonged activation contributes to maladaptive remodeling characterized by fibrosis and increased myocardial stiffness [146]. Evidence further suggests that ciliary TGF-β signaling in cardiac fibroblasts is required for fibrotic remodeling, linking primary cilia to pathological extracellular matrix deposition [140]. Pharmacological inhibition of TGF-β signaling during the fibrotic phase can reduce cardiac scarring and improve cardiac function in experimental models [147,148]. Conversely, several studies indicate that exogenous TGF-β1 may enhance cardiomyocyte regeneration and tissue repair, suggesting that controlled activation of this pathway could promote regenerative responses [149–151]. However, these findings remain inconsistent across models, underscoring the complexity of TGF-β signaling and the need for a deeper mechanistic understanding of its context-dependent effects [152].

An important challenge moving forward will be to determine how the primary cilium regulates TGF-β signaling dynamics across different cellular contexts and developmental stages. Addressing this question will require integrating advances in live-cell imaging, subcellular spatial proteomics, and genetic models to dissect how ciliary signaling modules control pathway activation, receptor trafficking, and downstream transcriptional responses in time and space. Such approaches may reveal how cilia selectively tune canonical and non-canonical TGF-β signaling outputs to shape cell fate decisions during development and regeneration.

Ultimately, understanding how the primary cilium coordinates TGF-β superfamily signaling may open new avenues for therapeutic intervention. By targeting cilia-dependent signaling mechanisms, it may become possible to modulate TGF-β activity with greater spatial and temporal precision, thereby promoting regenerative processes while limiting pathological remodeling. Continued investigation into this emerging signaling nexus promises to advance our understanding of developmental biology and may provide new strategies for treating cardiovascular, neurological, and fibrotic diseases.

## Perspectives

- **Highlight the importance of the field:** TGF-β superfamily signaling is central to tissue development and organ function, with the primary cilium emerging as a key regulator of its spatiotemporal dynamics.
- **Summary of the current thinking:** TGF-β superfamily signaling and the primary cilium are known to coordinate brain and heart development and homeostasis, but their mechanistic interplay is still not fully understood.
- **Comment on future directions:** Elucidating the mechanisms of cilia-dependent TGF-β superfamily signaling will advance fundamental understanding of ciliary signaling in development and homeostasis while enabling more precise modulation of these pathways for regenerative and therapeutic applications.

## Open Access

Open access for this article was enabled by the participation of the University of Copenhagen in an all-inclusive Read & Publish agreement with Portland Press and the Biochemical Society under an agreement with the Royal Danish Library.

## Abbreviations

ARL3: ADP-ribosylation factor-like protein 3
AURKA: Aurora kinase A
BBS: Bardet–Biedl syndrome
BMPs: bone morphogenetic proteins
CHD: congenital heart defects
CME: clathrin-mediated endocytosis
CvME: caveolin-mediated endocytosis
EMT: epithelial-mesenchymal transition
FGFR: fibroblast growth factor receptor
FHF: first heart field
GDFs: growth and differentiation factors
GPCRs: G protein-coupled receptors
Hh: Hedgehog
hESCs: human embryonic stem cells
IFT: intraflagellar transport
I-SMAD: inhibitor SMAD
MSC: mesenchymal stem cell
NPHP: nephronophthisis
PDGFRα: platelet-derived growth factor receptor alpha
R-SMADs: receptor-regulated SMADs
SHF: second heart field
TAK1: TGF-β-activated kinase 1
TGF-β: transforming growth factor-beta
